# Novel insights at the crossroads of antibiotic use and cancer risk

**DOI:** 10.15698/cst2023.06.280

**Published:** 2023-05-25

**Authors:** Nermina Malanovic, Djenana Vejzovic

**Affiliations:** 1 Institute of Molecular Biosciences, University of Graz, NAWI Graz, Graz, Austria.; 2 Field of Excellence BioHealth, University of Graz, 8010 Graz, Austria.; 3 BioTechMed Graz, 8010 Graz, Austria.; 4 Division of Biomedical Research, Core Facility Alternative Biomodels and Preclinical Imaging, Medical University of Graz, Graz, Austria.

**Keywords:** antibitiotics, cancer, immune cells, bacterial signature, antibacterial activity, anticancer activity, microbiome, drug resistance

## Abstract

The continuous use of antibiotics is associated with the spread of antimicrobial resistances and the not yet clear link to cancer development. Many conventional antibiotics have already shown different effects on a variety of cancer types raising questions for their rational use in cancer. However, discrepancy in the observed trend for some antibiotics reducing cancer development and being associated with higher risk of cancer underscores the lack of understanding the complex link between antibiotics and cancer. Here, we briefly summarize the possible antibiotic-mediated effects on cancer and conclude that those effects can be direct via i) specific targeting of tumor/cancer, ii) antimicrobial activity and iii) immunomodulatory activity whereby iv) indirectly caused effects primarily affect immune equilibrium between bacteria, cancer and immune cells. Furthermore, we also conclude that there is a great need for bulk profiling, comprehensive screening programs in all countries and in-depth studies to understand the risks and benefits of antibiotic use.

Fleming's discovery of the first natural antibiotic Penicillin in the late 1920's [1] opened a golden era for modern medicine enabling the control of bacterial infections [2]. In the 21^st^ century, however, their continuous use is associated with critical consequences that throw shadow on their success. These include the spread of antimicrobial resistances [3] and the not yet clear link to cancer development, as reports provide evidence for decreased survival of cancer patients exposed to antibiotics [4, 5]. Global action plans and stewardship programs to fight antimicrobial resistance led the global antibiotic market to decline in 2021 from $21 to $8 billion for on-patent antibiotics, however, this is obviously not resulting in lower antibiotic use as the global antibiotic market continues to shift towards lower-cost generics that may raise further serious concerns about inappropriate use [6].

Recent articles report a strong association of malignant tumors with fungi and bacteria, which exhibit high immuno-suppressive properties and strongly promote cancer progression [7–9]. Various cancer types appear to be associated with a unique microbiome residing inside the cancer with immune cells actively disturbing the immune equilibrium [7, 9–11]. Interestingly, decreased risk of rectal cancer was associated with past antibiotic use, most likely due to the anti-inflammatory impact of the used antibiotic tetracycline on a rectal inflammation associated with its oncogenesis [12–14]. Furthermore, researchers also point to growing evidence that an increased cancer risk arises more likely from an altered microbiome than from an inflammation in the gut. Also, treatment of mice bearing a colon cancer xenograft with the antibiotic metronidazole reduced the load of a cancer-colonized *Fusobacterium*, cancer cell proliferation, and overall tumor growth [15] questioning the innocent role of microbiota in cancer.

On the other hand, discrepancy in the observed trend for tetracyclines and macrolides reducing cancer development [16, 17] and being associated with higher risk of (breast) cancer [17, 18] underscores the lack of understanding the complex link between antibiotics and cancer. A recent study described the mechanism behind macrolide-induced tumor progression on melanoma and sarcoma *in vitro* and in mouse models *in vivo*, whereby macrolides block autophagic flux by inhibiting lysosomal acidification, inducing accumulation of radioactive species (ROS) and integrated stress response promoting tumor proliferation [19]. In patients, both breast and proximal colon cancer were linked to the use of other antibiotics including those targeting cell wall synthesis like penicillin or cephalosporins, but also to quinolones, sulfonamide and trimethoprim interfering with DNA and folate metabolism [12, 13, 20].

Apparently, there is no consensus, which antibiotic classes seem to be more associated with cancer prevalence. A summary by Gao and colleagues, discusses this double-edge sword of antibiotics used for cancer treatment and provides a rational basis for discussion of antibiotic use based on their modes of action [21]. Antibiotics in clinical use for bacterial infections can be classified according to the molecular mechanisms they use. A large majority of antibiotics target protein (tetracyclines, amphenicols, macrolides, aminoglycosides) and cell wall synthesis (glycopeptides, carbapenems, lipopeptides, phosphonates, cycloserines), but there are also antibiotics targeting DNA (azoles, fluoroquinolones), RNA (ansamycin, aipiarmycin) or their precursors through ATP or folate synthesis (sulfonamides) [22]. A common denominator for all those antibiotics is that although they inhibit a bacterial cell's elemental processes essential for life, they unfortunately do not eliminate the bacteria from the host body. Hence, the distinct microbial signature (RNA, DNA, proteins, endotoxins etc.) remains in the body of the host which may further interfere with cellular processes in the host, particularly inducing inflammation that may result in multi organ failure and sepsis/septic shock [23–26]. Although the release of bacterial endo(toxins) depends on antibiotic used, the trend for rapid clinical deterioration increases [25]. In addition, it is also believed that bacteria by producing diverse proteins and enzymes prevent immune cells from killing cancer and prevent cancer cells from drug treatment and hence, are responsible for drug resistance [9, 10]. In this context, it was shown in mice that tumors develop resistance to the chemotherapeutic drug gemcitabine in the presence of bacteria, which was reversed by antibiotic treatment [10]. Authors also point to the potential of antibiotic adjunctive treatment to cancer therapy.

Many conventional antibiotics have already shown different effects (**Figure 1**) on a variety of cancer types raising questions for their rational use in cancer [14, 17, 27, 28]. However, the question arises whether antibiotics should be reconsidered as anticancerogenic, too. Indeed, some antibiotics showed anticancer effects independent of their antibacterial effects. Examples include flavoxin antibiotics which intake was beneficial for patients with lung and bladder cancer and flavoxin antibiotics were identified to inhibit p90 ribosomal protein S6 kinase 4 (RSK4), a common regulator of chemosensitivity in bladder and lung cancer [29].

**Figure 1:**
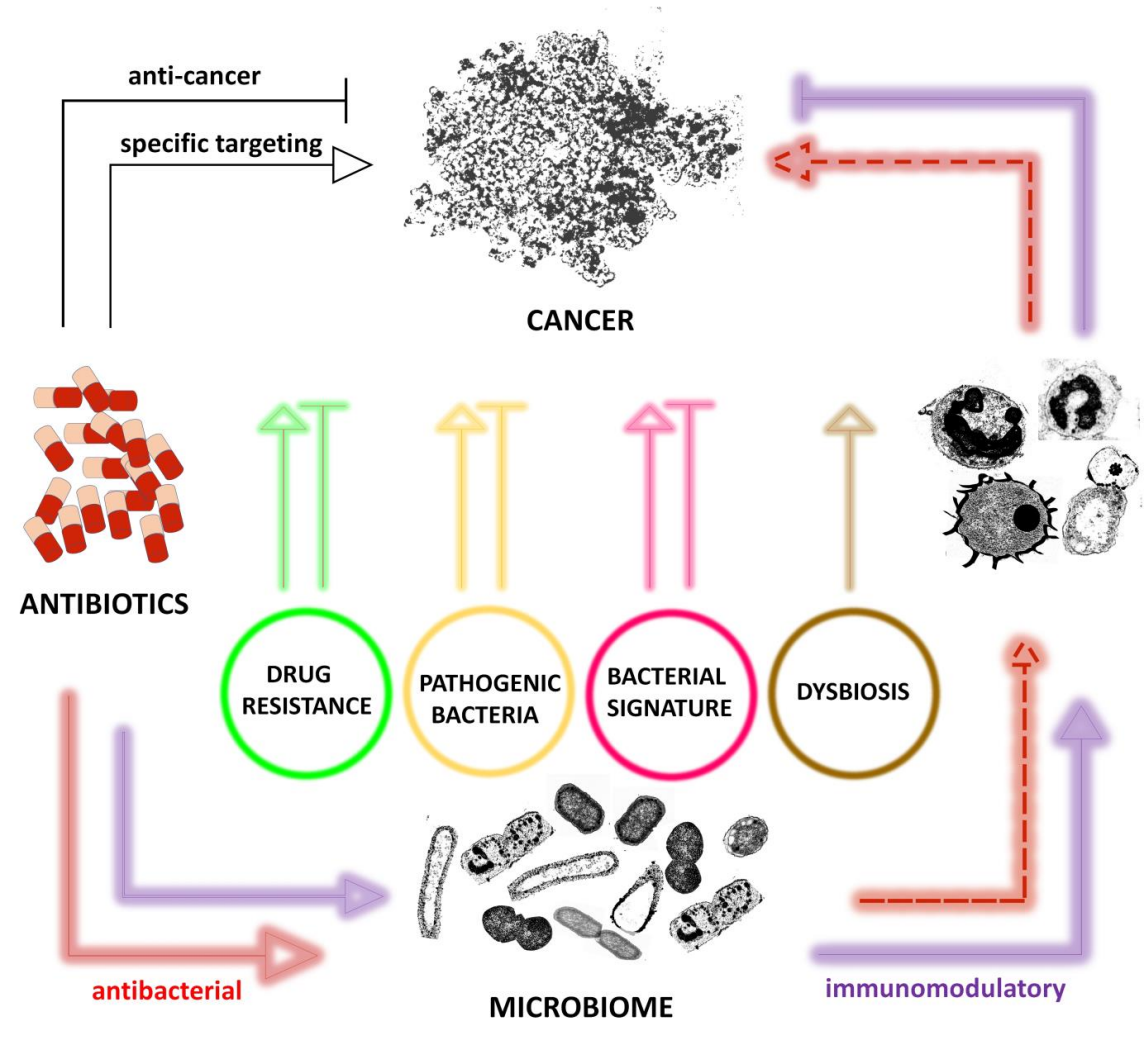
FIGURE 1: Possible effects of antibiotics on cancer. Antibiotic-mediated effects on cancer are illustrated according to their direct effects on cancer via specific targeting (black line), antibacterial activity (red line) and immunomodulatory activity (purple line), with arrows indicating activating and blunt ended lines indicating anticancer effects. However, some effects are indirectly (dashed line) linked to cancer, as they have a strong impact on the immune response and thus on the immune equilibrium between bacteria, cancer and immune cells. It should also be noted that it is not clear yet wheather some of the effects are due to antibiotics, bacteria themselves or bacterial products released independently of antibiotics or upon antibiotic exposure.

Although the link between antibiotics and cancer is growing, in discussion with renowned scientists, there is clear evidence that there is a great need for bulk profiling, comprehensive screening programs in all countries and in-depth studies to understand the risks and benefits of antibiotic use [13].
